# Vitamin D Receptor Polymorphisms Are Associated with 25(OH)D_3_ Concentrations and Systemic Inflammation in Decompensated Cirrhosis

**DOI:** 10.3390/nu18193234

**Published:** 2026-09-30

**Authors:** Alina Bauschen, Mona-May Langer, Helena Stadler, Nicholas Zeuzem, Birte Möhlendick, Sabrina Guckenbiehl, Christian M. Lange

**Affiliations:** 1 Department of Internal Medicine I, University Medical Center of the Johannes Gutenberg University Mainz, 55131 Mainz, Germany; alina.bauschen@unimedizin-mainz.de (A.B.); nicholas.zeuzem@unimedizin-mainz.de (N.Z.); 2 Department of Internal Medicine II, LMU University Hospital, 81377 Munich, Germany; 3 Department for Gastroenterology and Hepatology, University Hospital Essen, University Duisburg-Essen, 45147 Essen, Germany; 4 Institute for Pharmacogenetics, University Hospital Essen, University Duisburg-Essen, 45147 Essen, Germany

**Keywords:** liver cirrhosis, acute-on-chronic liver failure, vitamin D, vitamin D receptor, single nucleotide polymorphisms, systemic inflammation

## Abstract

**Background/Objectives**: Vitamin D regulates both the adaptive and the innate immune system, while having anti-inflammatory, immunomodulatory and anti-fibrotic properties. In clinical studies, 25(OH)D_3_ deficiency was associated with systemic inflammation, but it is unclear whether these associations are causal. In this study, we aimed to investigate the impact of Vitamin D receptor (VDR) polymorphisms (SNPs)—as surrogates for functional relationships—on 25(OH)D_3_ status and systemic inflammation in decompensated liver cirrhosis. **Methods**: Patients with compensated and decompensated cirrhosis with or without ACLF were enrolled prospectively from a monocentric cohort study (N = 355). Pyrosequencing was performed for genotyping SNPs (*rs7968585*, *rs731236 (TaqI)*, *rs7975232 (ApaI)*, *rs2239179* and *rs2228570 (FokI)*). VDR-SNPs were analysed for their association with 25(OH)D_3_ concentrations and markers of systemic inflammation. **Results**: Severe 25(OH)D_3_ deficiency was frequent in decompensated cirrhosis. 25(OH)D_3_ supplementation was associated with significantly higher circulating 25(OH)D_3_ in compensated and decompensated liver cirrhosis, whereas in ACLF, 25(OH)D_3_ levels did not differ significantly between supplemented and unsupplemented patients. An inverse correlation of 25(OH)D_3_ and IL-6 levels was identified (R = −0.2286, 95% CI −0.328 to −0.124, *p* < 0.0001). In exploratory analyses among the analyzed VDR-SNPs, the GG-variant of *rs2239179* was associated with lower 25(OH)D_3_ levels (β = 0.705, 95% CI 0.498-0.998, *p* = 0.049). Moreover, the functionally important VDR-SNP *rs2228570 TC*-variant showed a nominal independent association with higher IL-6 levels (ß = 1.685, 95% CI 1.102–2.576, *p* = 0.016) after adjustment. **Conclusions**: These exploratory findings suggest that VDR genetics may partially contribute to 25(OH)D_3_ status and systemic inflammation in liver cirrhosis and ACLF and warrant further replication in independent cohorts.

## 1. Introduction

Liver cirrhosis is a major global health burden, with an estimated 112 million people living with compensated and 10.6 million with decompensated cirrhosis worldwide. Liver disease is responsible for roughly two million deaths annually [1]. Beyond its epidemiological burden, liver cirrhosis is associated with substantial healthcare utilization due to current hospitalizations for decompensating events, and liver transplantation remains the only curative option for most patients—underscoring the clinical need for strategies that target modifiable drivers of disease progression, such as systemic inflammation and micronutrient status [2].

Liver cirrhosis represents the end stage of chronic liver disease. Compensated liver cirrhosis is defined by absence of cirrhosis-associated complications such as infections, hepatic encephalopathy, ascites and gastrointestinal bleedings. With disease progression and transition from compensated to decompensated cirrhosis, liver related mortality does strongly increase [3]. If additional specific organ failures occur, acute-on-chronic liver failure (ACLF) develops, a distinctive syndrome associated with a high short-time mortality [4]. Overall, liver insufficiency, systemic inflammation and portal hypertension are referred to as major factors in the development of decompensation and ACLF [5].

Pleiotropic factors are contributing to the frequent development of 25(OH)D_3_ deficiency in chronic liver disease [6]. This is mainly due to decreasing liver cell biomass resulting in a diminished capacity of hydroxylating enzymes. Simultaneously, patients are suffering from reduced sunlight exposure, limited intestinal absorption capacity and decreasing albumin and 25(OH)D_3_ binding protein synthesis [7]. A substantial body of evidence links 25(OH)D_3_ deficiency to hepatic decompensation, systemic inflammation and adverse outcomes across chronic liver disease etiologies. Low 25(OH)D_3_ levels are not merely a consequence of chronic liver disease but also appear to directly participate in pathological processes in liver cirrhosis [7,8]. 25(OH)D_3_ is a steroid-hormone that is classically involved in calcium and phosphate homeostasis and bone metabolism [6]. In addition, various non-classical functions have been described. Of note, by signaling through Vitamin D receptor (VDR), a nuclear transcription factor, 25(OH)D_3_ serves as a modulator of adaptive and innate immune functions [9,10]. VDR is among others expressed by circulating immune cells, Kupffer cells and hepatic stellate cells (HSCs) and helps to downregulate pro-inflammatory signals while simultaneously upregulating anti-inflammatory mediators [11]. Moreover, antifibrotic properties are demonstrated. 25(OH)D_3_ inhibits HSC activation and consequently fibrogenesis by impeding TGFβ/SMAD signaling [12,13]. On top of these, 25(OH)D_3_ supports the intestinal epithelial barrier integrity, thereby reducing bacterial translocation and decompensation risk [14].

Within VDR, genetic variations are frequently found. Common single nucleotide polymorphisms (SNPs) are *FokI*, *TaqI* and *ApaI* [15]. *FokI* shortens the VDR protein by alternating the start codon. *TaqI* causes a silent mutation and influences messenger ribonucleic acid (mRNA) stability without changing the protein sequence itself. Likewise, *ApaI* affects mRNA stability and splicing [16,17].

Despite the above-mentioned well-established association, existing supplementation trials in chronic liver disease have yielded heterogenous results without consistent benefit on survival or hepatic fibrosis and steatosis markers and inflammation [18]. No evidence-based, cirrhosis-specific supplementation strategies exist, and it remains unclear which patients are likely to respond to supplementation, and which are not. A key reason for this uncertainty is that the interplay of 25(OH)D_3_, VDR genotype and inflammation in the context of liver cirrhosis and ACLF remains incompletely understood. Clarifying the determinants of 25(OH)D_3_ status and its relationship to systemic inflammation represents a clinically relevant and currently unmet need in the management of liver cirrhosis. This study therefore aimed to investigate the independent contribution of VDR genetics to 25(OH)D_3_ status, supplementation and systemic inflammation across the full spectrum of liver cirrhosis, including ACLF.

## 2. Materials and Methods

### 2.1. Patients

Patients that were hospitalized with liver cirrhosis at the University Hospital Essen, Germany and liver cirrhosis patients presenting to the outpatient liver clinic of the University Hospital Essen between December 2018 and May 2022 were included in this prospective cohort study (N = 355). Buffy coats of healthy donors (N = 22) were provided by the Institute for Transfusion Medicine of the University Hospital Essen and used as reference for IL-6 concentrations. These were anonymized routine donations. No demographic or clinical data were available, and matching cirrhosis cohort was therefore not possible.

Written informed consent was obtained from all participants prior to enrollment. Ethical approval was secured from the local ethical committee of the University Duisburg-Essen (WBE-071, approval date 3 September 2018). Exclusion criteria were refusal of participation, pregnancy, breastfeeding, being below the full legal age, presence of hepatocellular carcinoma beyond Milan criteria, as well as positive testing for Human Immunodeficiency Virus. Samples were collected at baseline, and clinical and laboratory values were recorded accordingly.

25(OH)D_3_ supplementation was recorded as a binary variable at baseline. Information on dose, duration, adherence and interval between intake and blood sampling was not systematically available.

ACLF was classified based on the criteria of the Chronic Liver Failure—European Association for the Study of the Liver (CLIF-EASL) consortium. Decompensation was defined according to the criteria of the CANONIC study and the EASL-CLIF consortium [19] as the presence of at least one of the following: gastrointestinal hemorrhage, bacterial infection, new onset/progression of hepatic encephalopathy (graded by West-haven criteria) or ascites. No patient was classified as decompensated based on minimal ascites alone.

### 2.2. Quantification of 25-Hydroxyvitamin D (25(OH)D_3_) Plasma Levels

25(OH)D_3_ was determined in plasma samples. 25(OH)D_3_ concentrations were quantified by ADVIA Centaur Vitamin D Total Assay (Siemens, Healthengineers AG, Forchheim, Germany) in the central laboratory of the University Hospital Essen, Germany. 25(OH)D_3_ levels below 10 ng/mL were considered as a severe deficiency, whereas 25(OH)D_3_ levels of 10–20 ng/mL and 20–30 ng/mL were referred to as a mild deficiency and insufficiency, respectively. Sufficient 25(OH)D_3_ plasma concentrations were defined as being above 30 ng/mL [20,21].

### 2.3. Quantification of Interleukin-6 (IL-6) Plasma Levels

Plasma concentrations of IL-6 were quantified by ELISA. Samples were measured undiluted with a commercial ELISA assay kit (Quantikine^®^ ELISA Human IL-6 Immunoassay, R&D Systems, Minneapolis, MN, USA) according to the manufacturer’s instructions. The optical density of each well was determined within 30 min using a microplate reader (FluoStar Omega V5.50, BMG Labtech, Ortenberg, Germany) set to 450 nm with wavelength correction at 540 nm and 570 nm.

### 2.4. Density Gradient Centrifugation

Density gradient centrifugation was performed to isolate granulocytes and human peripheral blood mononuclear cells (PBMCs). Blood was diluted with phosphate-buffered saline (PBS) and layered onto PANcoll (Pan Biotech, Aidenbach, Germany). Centrifugation of layered PANcoll was performed without brake at 4 °C, 600× *g* for 15 min. Then, cells were aspirated from the corresponding layer and washed twice with PBS for 10 min at 4 °C and 300× *g*. Cells were finally resuspended in 1 mL freezing medium (90%FCS + 10% DMSO) and stored at −80 °C.

### 2.5. Deoxyribonucleic Acid (DNA) Extraction and Quantification

Genomic DNA was extracted from granulocytes isolated as described above. DNA was purified using the QIAmp DNA Mini Kit (Quiagen, Hilden, Germany) according to the manufacturer’s protocol. Concentration and quality of extracted DNA were assessed by Implen NanoPhotometer^®^ N60 (Implen, Munich, Germany).

### 2.6. Pyrosequencing of the Vitamin D Receptor (VDR) Gene for Single Nucleotide Polymorphism (SNP)

The VDR gene was genotyped for the SNPs *rs7968585*, *rs731236 (TaqI)*, *rs7975232 (ApaI)*, *rs2239179* and *rs2228570 (FokI)* by pyrosequencing. Pyrosequencing was performed using the PyroMark Q96 MD (Quiagen, Hilden, Germany) at the Institute of Pharmacogenetics, University Duisburg-Essen, Germany. For genotyping, 500 ng of DNA were used.

### 2.7. Statistical Analysis

Statistical analysis was performed using GraphPad Prism V11.0.1 (GraphPad Software, San Diego, CA, USA) and Jamovi v2.7.30 (Jamovi Software, Sydney, Australia). The analyses were conducted at a two-sided significance level. *p* values less than 0.05 were considered statistically significant. After testing for Gaussian distribution, two groups were compared by T-test or Wilcoxon–Mann–Whitney U-test, as appropriate. Comparison of more groups was done by one-way ANOVA or the Kruskal–Wallis test. Categorial data were analyzed by X^2^ test. Spearman’s rank correlation was used to assess associations between continuous variables. Simple linear regression was performed for univariate analysis of continuous outcomes, and variables with *p* < 0.05 were considered for multivariate modelling. Multicollinearity was assessed using VIF (all < 5). Patients with missing values for any included covariate were excluded from the respective model without imputation, with missingness assumed to be at random. Effect estimates from regression and correlation analyses are reported with 95% confidence intervals. Group comparisons are reported as measures of central tendency and dispersion with corresponding *p* values.

The linkage equilibrium, haplotype analyses and calculation of Hardy–Weinberg equilibrium were performed by the Institute for Pharmacogenetics, University Duisburg-Essen, Germany using Haploview 4.3 (Broad Institute, Cambridge, MA, USA). Five VDR variants were selected a priori on the basis of established functional relevance rather than screened in an agnostic manner, and three of them were in high linkage disequilibrium and were therefore analyzed as a single haplotype. As the tests performed were thus neither independent nor hypothesis-free, a genome-wide-style correction for multiple comparisons would be excessively conservative in this setting. We therefore report nominal *p* values throughout and interpret all genotype associations as exploratory and hypothesis-generating. The possibility of false-positive findings cannot be excluded, and an independent replication is required before any of these associations can be regarded as established.

## 3. Results

### 3.1. Baseline Characteristics

A total of 355 patients with liver cirrhosis were included in the present study. Among those, 163 (45.9%) patients had compensated liver cirrhosis, whereas 161 (45.4%) presented with decompensated liver cirrhosis. A total of 31 (8.7%) patients were diagnosed with ACLF. Alcohol-related liver cirrhosis was the most common cause of decompensated cirrhosis (N = 77, 47.83%), as well as of ACLF (N = 24, 77.4%), while autoimmune hepatitis was the leading aetiology in compensated cirrhosis (N = 49, 30%). Commonly used severity and prognostic scores worsened along the disease spectrum from compensated to decompensated stage and further to ACLF (Child Pugh comp. vs. ACLF: *p* < 0.0001; ReMELD-Na comp. vs. ACLF *p* < 0.0001). Detailed baseline characteristics of included patients are shown in Table 1.

### 3.2. 25(OH)D_3_ Deficiency Is Highly Prevalent and 25(OH)D_3_ Supplementation Effectively Improves 25(OH)D_3_ Status in Liver Cirrhosis

In the overall cohort, mean 25(OH)D_3_ plasma levels were 15.3 ng/mL. A total of 120 patients (33.8%) were 25(OH)D_3_ supplemented, while 235 (66.2%) did not receive any supplementary therapies (Table 1). Mean 25(OH)D_3_ concentrations of supplemented and non-supplemented patients were quantified at 20.2 ng/mL and 12.8 ng/mL, respectively (*p* < 0.0001). In further multivariate regression adjusting for VDR genotypes and cirrhosis severity, 25(OH)D_3_ supplementation remained an independent predictor of higher 25(OH)D_3_ levels (β = 1.497, 95% CI 1.297–1.729, *p* < 0.001) (Supplementary Table S2).

### 3.3. 25(OH)D_3_ Levels Decrease with Increasing Severity of Liver Cirrhosis and Do Not Differ Significantly by Supplementation Status in ACLF

**Figure 1:**
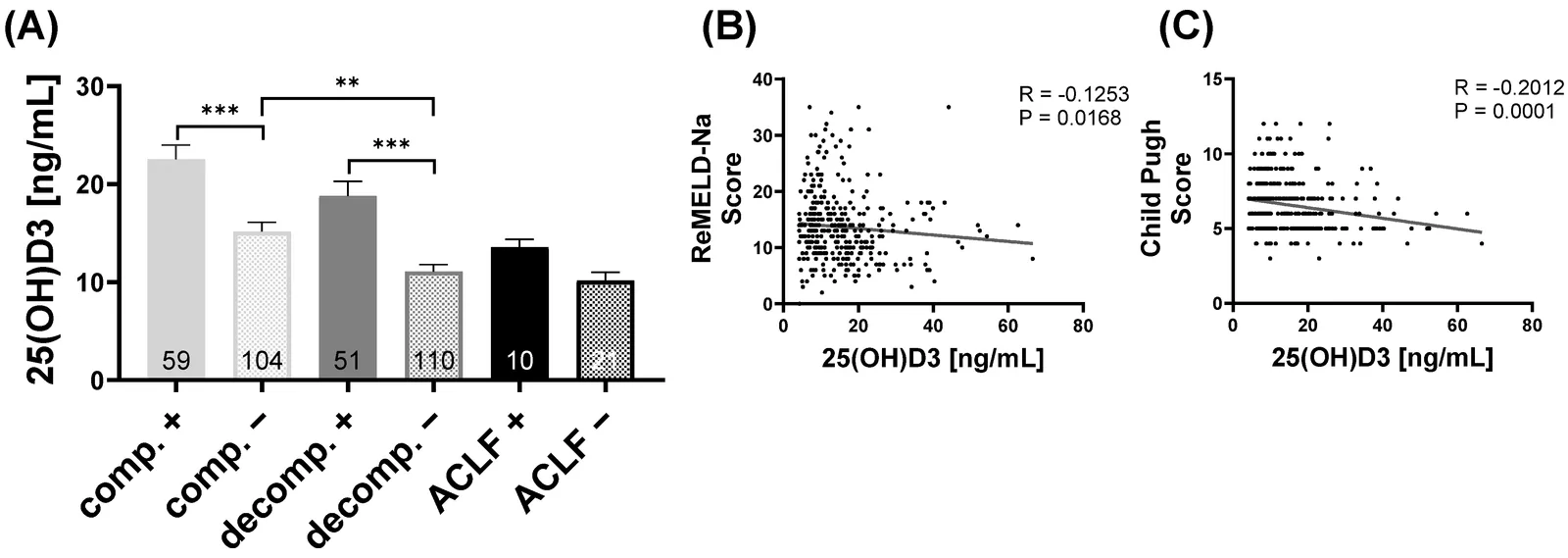
(**A**) Bar charts showing mean 25(OH)D_3_ concentrations of compensated (comp.), decompensated (decomp.) and ACLF patients with (+) and without (−) 25(OH)D_3_ supplementation. Associations of 25(OH)D3 levels and ReMELD-Na Score (**B**) as well as Child Pugh Score (**C**) were assessed using Spearman’s rank correlation. Corresponding correlation coefficients (R) and *p*-values are presented in the figure. Data are presented as mean with SEM. Statistical significance was determined by one-way ANOVA or Kruskal–Wallis test, as appropriate. ** *p* ≤ 0.01, *** *p* ≤ 0.001.

The subgroups of liver cirrhosis did not differ significantly with respect to the proportion of patients receiving 25(OH)D_3_ supplementation (comp. vs. decomp.: *p* = 0.4; comp. vs. ACLF: *p* = 0.7; decomp. vs. ACLF: *p* = 0.9) (Table 1). Mean 25(OH)D_3_ concentrations in compensated and decompensated liver cirrhosis were 17.8 ng/mL and 13.55 ng/mL, respectively (*p* < 0.0001). Among all groups, 25(OH)D_3_ levels were lowest in ACLF (11.3 ng/mL; *p* = 0.0068) (Figure 1A). 25(OH)D_3_ showed significant inverse correlation with both ReMELD-Na Score (R = −0.1253, 95% CI −0.230 to −0.018, *p* = 0.0168) and Child Pugh Score (R = −0.2012, 95% CI −0.299 to −0.099, *p* = 0.0001) (Figure 1B,C). However, in multivariate analyses, ascites rather than ReMELD-Na Score emerged as the dominant independent predictor of 25(OH)D_3_ status (β = 0.747, 95% CI 0.609–0.917, *p* = 0.005), suggesting the clinical manifestation of hepatic decompensation is the determinant of deficient 25(OH)D_3_ metabolism rather than laboratory-based quantification of cirrhosis severity (Supplementary Table S2).

25(OH)D_3_ concentrations were significantly higher in supplemented patients in both compensated (22.5 ng/mL vs. 15.3 ng/mL: *p* < 0.0001) and decompensated liver cirrhosis (18.8 ng/mL vs. 11.1ng/mL: *p* < 0.0001). Despite that, in ACLF, 25(OH)D_3_ levels did not differ significantly by supplementation status (13.5 ng/mL vs. 10.1 ng/mL; *p* > 0.9) (Figure 1A). Whether this reflects reduced responsiveness in ACLF mediated by systemic inflammation overwhelming exogenous 25(OH)D_3_ or simply differences in dose, duration, adherence or timing of supplementation cannot be determined from these data.

Figure 1 25(OH)D_3_ plasma levels decrease with progressing liver cirrhosis and show no significant differences by supplementation in ACLF.

### 3.4. 25(OH)D_3_ Status Is Inversely Associated with IL-6 Levels but Not an Independent Predictor of Systemic Inflammation in Liver Cirrhosis

**Figure 2:**
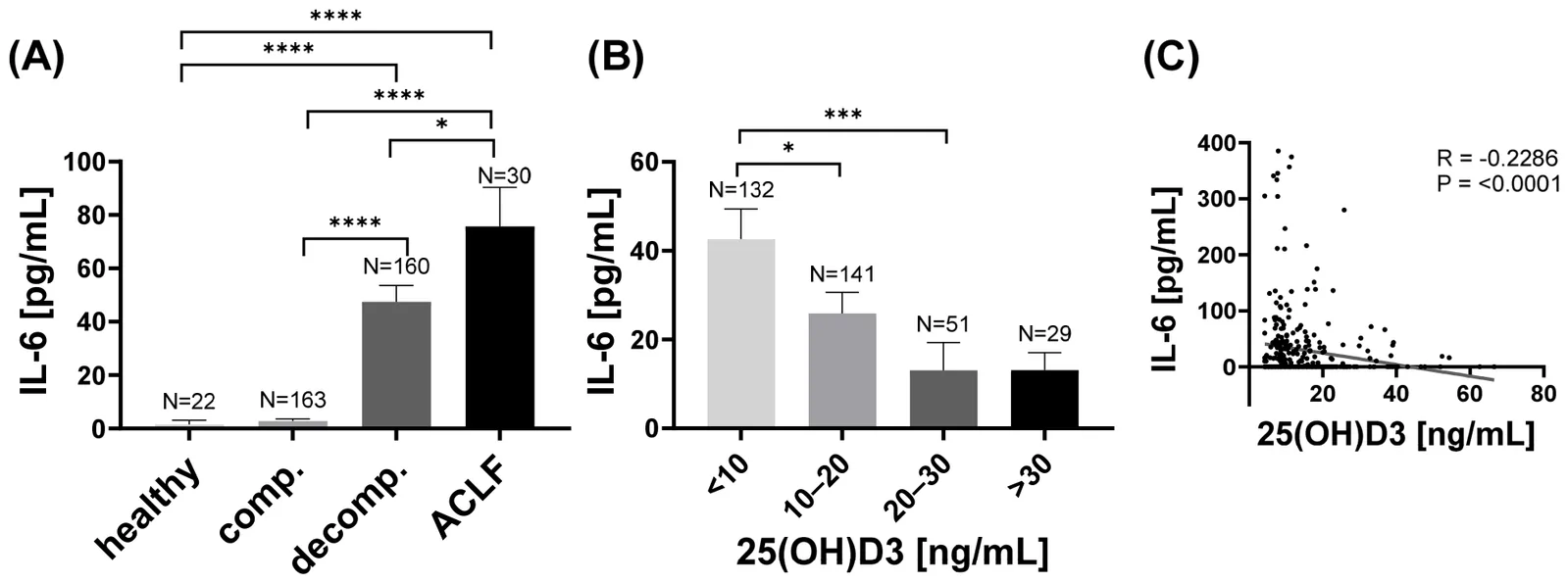
(**A**) Bar charts showing mean IL-6 concentrations in healthy, compensated (comp.), decompensated (decomp.) and ACLF patients as well as (**B**) in 25(OH)D_3_ reference value subgroups. (**C**) Association of IL-6 and 25(OH)D_3_ levels was assessed using Spearman’s rank correlation. Corresponding correlation coefficients (R) and *p*-values are presented in the figure. Data are presented as mean with SEM. Statistical significance was determined by one-way ANOVA or Kruskal–Wallis test, as appropriate. * *p* ≤ 0.05, *** *p* ≤ 0.001, **** *p* ≤ 0.0001.

In healthy controls and compensated liver cirrhosis, mean IL-6 plasma levels were quantified at 1.6 pg/mL and 2.8 pg/mL, respectively (*p* > 0.9). Progression towards decompensated cirrhosis was associated with a significant increase in IL-6 (mean 47.5 pg/mL, median 17.19 ng/mL [IQR 9.00–53.45]; *p* < 0.0001). Ultimately, the highest concentrations were determined in ACLF (mean 75.73 ng/mL, median 46.70 ng/mL IQR [5, 28, 37–111]; comp. vs. ACLF: *p* < 0.0001; decomp. vs. ACLF: *p* = 0.01) (Figure 2A). In patients with sufficient 25(OH)D_3_ status, IL-6 concentrations were measured lowest. With increasing 25(OH)D_3_ deficiency, IL-6 rose significantly (Figure 2B). Consistent with these findings, Spearman’s rank correlation revealed a significant inverse correlation of 25(OH)D_3_ and IL-6 levels (R = −0.2286; 95% CI −0.328 to −0.124, *p* < 0.0001) (Figure 2C). Further univariate linear regression analysis confirmed 25(OH)D_3_ as a significant predictor of IL-6 levels (β = 0.965, 95% CI 0.946–0.984, *p* = 0.0004). Nevertheless, this association did not remain independent after adjusting for markers of disease severity (Table 2). These results suggest 25(OH)D_3_ not to be an independent driver of IL-6 level in cirrhosis. The association may be modulated by disease severity rather than reflect a direct independent causal relationship. Given these findings, we next aimed to investigate how genetic VDR variants modulate this relation.

Figure 2 IL-6 plasma levels increase with liver cirrhosis progression and show inverse association with 25(OH)D_3_ status.

### 3.5. The rs2239179 GG-Variant Shows Association with 25(OH)D_3_ Status in the Overall Cohort

When analyzing VDR-SNP distribution and linkage disequilibrium (LD) of VDR-SNPs in our cohort, we identified SNPs *rs731236*, *rs7975232*, *rs7968585* to be in high LD (Supplementary Table S1). These findings were consistent with published data [22]. Based on this, we went on summarizing these SNPs as a haplotype (Supplementary Table S1). Hardy–Weinberg equilibrium was assessed for all variants in the overall cohort (Supplementary Table S1). rs7968585 and rs7975232 deviated from equilibrium (*p* = 0.0001 and *p* = 0.0002, respectively), both showing an excess of heterozygotes, whereas all other SNPs investigated were in equilibrium. Overall, no significant differences in the prevalence of different genotypes of haplotype *rs2239179* and *rs2228570* were observed in different stages of liver cirrhosis (Table 3).

**Figure 3:**
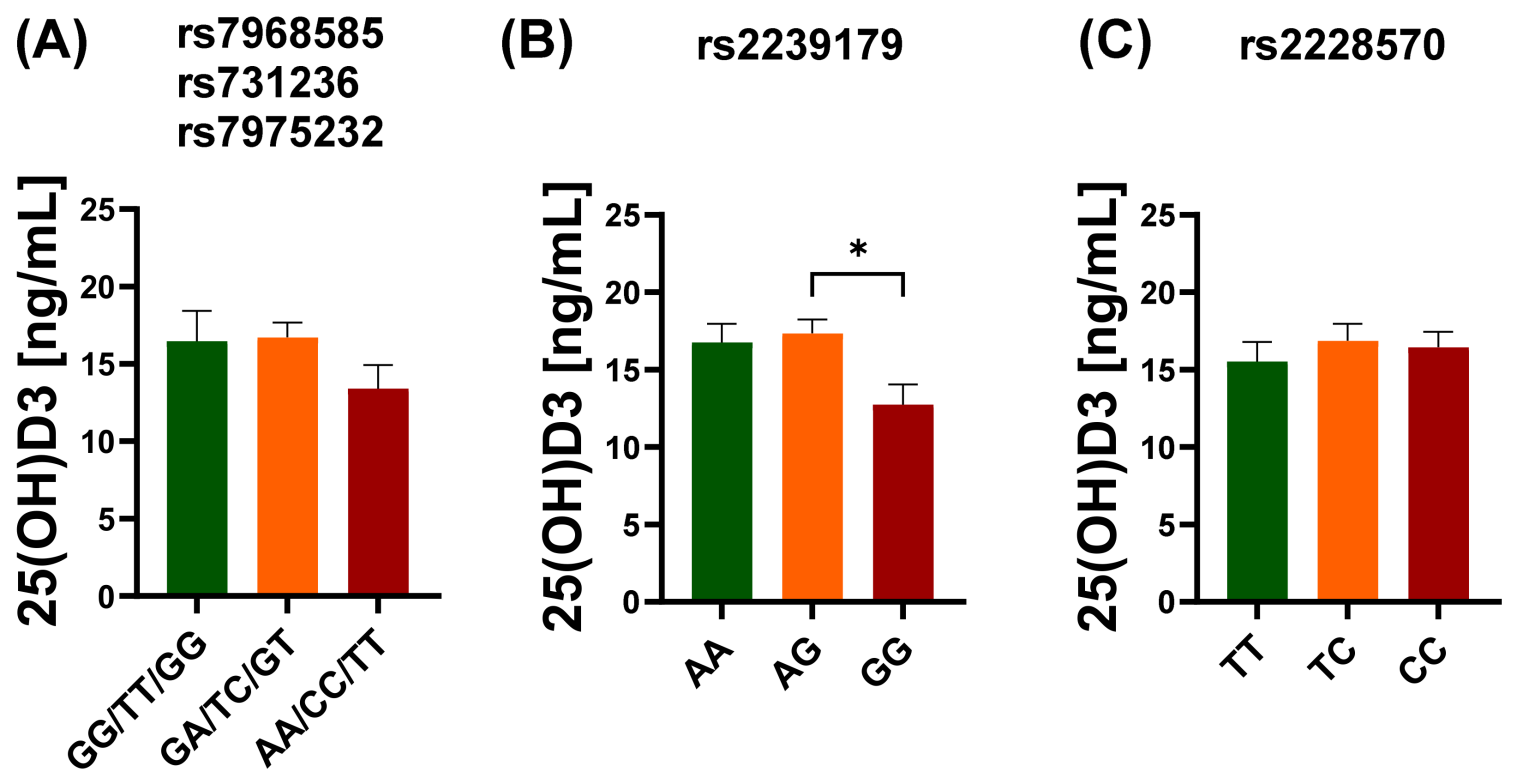
Bar graphs show 25(OH)D_3_ concentrations of the overall cohort according to (**A**) haplotype 7968585/rs731236/rs7975232, (**B**) rs2239179 and (**C**) rs2228570. Wildtype, heterozygous and homozygous variants are shown in green, orange and red, respectively. Data are presented as mean with SEM. Statistical significance was determined by one-way ANOVA or Kruskal–Wallis test, as appropriate. * *p* ≤ 0.05.

Next, the effect of VDR-SNPs on 25(OH)D_3_ status was assessed. In Kruskal–Wallis analysis among all investigated SNPs, only *rs2239179* was significantly associated with 25(OH)D_3_. Individuals carrying the homozygous GG-variant showed lower 25(OH)D_3_ levels compared to AG carriers (12.74 ng/mL vs. 17.7 ng/mL: *p* = 0.02) (Figure 3B). Since these analyses reflect comparison of unadjusted raw values, we further performed uni- and multivariate regression analyses using wildtype variants as reference. Here, both heterozygous (β = 1.18, 95% CI 1.02–1.366, *p* = 0.026) and homozygous (β = 0.795, 95% CI 0.651–0.97, *p* = 0.024) haplotypes showed significant univariate association with 25(OH)D_3_. Moreover, *rs2239179* GG-variant exhibited significantly lower 25(OH)D_3_ levels as compared to AA carriers (β = 0.792, 95% CI 0.66–0.950, *p* = 0.012). In multivariate analysis, GG-variant of *rs2239179* remained a nominal independent predictor of 25(OH)D_3_ status (β = 0.705, 95% CI 0.498–0.998, *p* = 0.049) (Supplementary Table S2).

Figure 3 25(OH)D_3_ plasma levels are associated with the corresponding genotype of VDR-SNP rs2239179 in the overall cohort.

### 3.6. VDR-SNP rs2228570 Showed a Nominal Independent Association with IL-6 Level in Liver Cirrhosis

**Figure 4:**
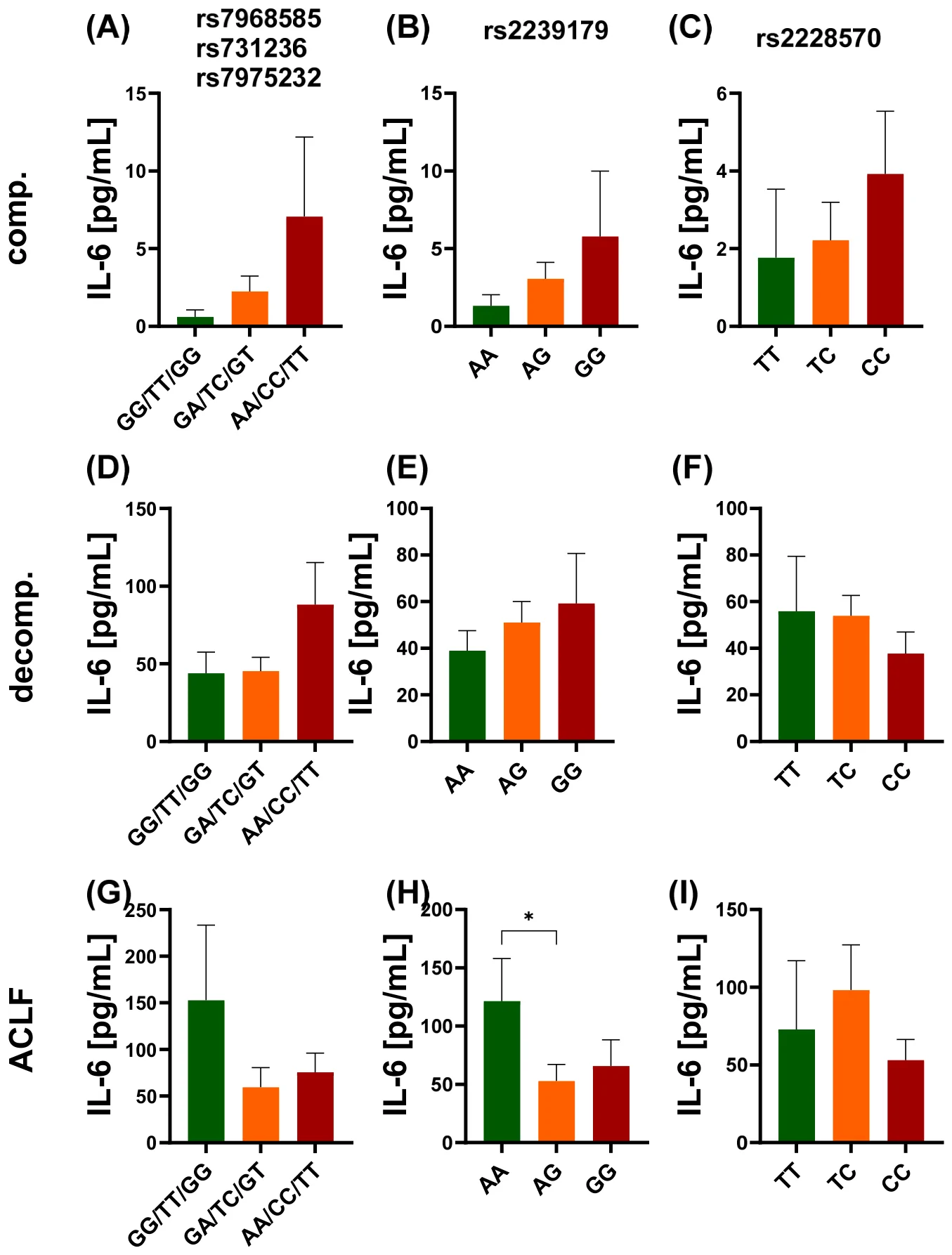
Bar graphs show IL-6 concentrations in compensated (comp.; (**A**–**C**)), decompensated (decomp.; (**D**–**F**)) liver cirrhosis and ACLF (**G**–**I**) according to VDR-SNPs. Group sizes per panel were: (**A**) N = 35, 63, 18; (**B**) N = 57, 84, 22; (**C**) N = 29, 69, 65; (**D**) N= 31, 66, 19; (**E**) N = 60, 81, 20; (**F**) N = 17, 79, 65; (**G**) N = 4, 13, 3; (**H**) N = 10, 19, 2; (**I**) N = 5, 13, 13. Given the small size of ACLF group, further divided by genotype, ACLF-stratified comparisons (**G**–**I**) should be regarded as exploratory. Wildtype, heterozygous and homozygous variants are shown in green, orange and red, respectively. Data are presented as mean with SEM. Statistical significance was determined by one-way ANOVA or Kruskal–Wallis test, as appropriate. * *p* ≤ 0.05.

Given the substantial different inflammatory milieu across the subgroups of liver cirrhosis, stratified analyses were chosen to analyze VDR-SNP effects on IL-6 concentrations. This approach further accounts for potential effect modification by disease stage and avoids masking genotype-specific effects, which may occur in pooled analyses. In both compensated and decompensated liver cirrhosis, a consistent trend towards higher IL-6 concentrations in AA/CC/TT carriers compared to GG/TT/GG carriers was found (comp. 0.61 pg/mL vs. 7.07 pg/mL; *p* = 0.99; decomp. 43.0 pg/mL vs. 88.21 pg/mL; *p* = 0.767) (Figure 4A,D). Likewise, in *rs2239179* a tendency towards higher IL-6 in GG-variant as compared to AG- and AA-variants was observed (comp. 1.32 pg/mL vs. 5.78 pg/mL; *p* = 0.99; decomp. 38.92 pg/mL vs. 59.24 pg/mL; *p* = 0.99) (Figure 4B,E). Notably, in ACLF *rs2239179* AA-variant showed significantly higher IL-6 levels compared to heterozygous individuals (121.5 pg/mL vs. 53.0 pg/mL: *p* = 0.04) (Figure 4H). Given the small subgroup sizes, these analyses were underpowered and are reported as exploratory.

To further isolate the independent contribution of VDR-SNPs to IL-6 levels, another multivariate model including VDR genetic was performed. In line with the first model (Table 2) 25(OH)D_3_ remained non-significant (*p* = 0.763). In multivariate analysis the TC-variant of *rs2228570* showed a nominal independent association with higher IL-6 levels as compared to wildtype carriers in the overall cohort (ß = 1.685, 95% CI 1.102–2.576, *p* = 0.016) (Table 4). What is more, inclusion of *rs2228570* in the multivariate IL-6 model (Table 4, R^2^ = 0.604) modestly improved the model fit when compared to the clinical base model (Table 2; R^2^ = 0.589)—an increment in line with what is expected for a single common variant contributing to a multifactorial phenotype, where the relevance lies in identifying a genetic contribution to inflammatory burden rather than in improving prediction.

Collectively, these findings should be regarded as exploratory, suggesting that VDR genetics may contribute to systemic inflammation independently of 25(OH)D_3_, a hypothesis requiring confirmation in independent cohorts.

Figure 4 The AA-variant of rs2239179 may be associated with higher IL-6 levels in ACLF.

## 4. Discussion

In the present study we provide a comprehensive analysis of the associations between VDR-SNPs, 25(OH)D_3_ status, 25(OH)D_3_ supplementation and inflammation in a well-characterized cohort, including ACLF as a newly explored entity in this context. In line with recent data, we could highlight liver cirrhosis as a condition of vulnerability to 25(OH)D_3_ deficiency and re-emphasize its aggravation with progressing liver cirrhosis due to loss of liver cell biomass [8,23,24]. This progressive 25(OH)D_3_ decline paralleled the expected worsening of clinical complications and routine laboratory and organ-function parameters across compensated, decompensated and ACLF groups, as detailed in Table 1.

25(OH)D_3_ supplementation was associated with higher 25(OH)D_3_ concentrations in compensated and decompensated cirrhosis, but not in ACLF. Therefore, it proved to be an effective approach to raise 25(OH)D_3,_ suggesting exogenous 25(OH)D_3_ may overcome the impaired endogenous hydroxylation across the broader cirrhosis population. As this is an observational cohort in which supplementation was recorded only as a binary variable, we cannot determine whether this reflects reduced biological responsiveness from differences in dose, duration, adherence or timing of supplementation. ACLF represents a hyperinflammatory milieu [25,26] that may plausibly interfere with 25(OH)D_3_ metabolism such that additional supplementation fails—a hypothesis for prospective, dose-controlled studies rather than an established finding.

Previously, direct immunomodulatory properties of 25(OH)D_3_ were described. For example, via VDR signaling, 25(OH)D_3_ increases anti-inflammatory and decreases pro-inflammatory cytokine production [27]. It promotes differentiation of helper T cells 1 (T_H_1) to T_H_2 cells, increases T_H_2 cells as well as regulatory T cell (T_regs_) activity and has inhibitory effects on naïve CD4+ T cells, resulting in less T_H_17 cell differentiation. Additionally, it enhances innate immune cell function [27,28,29]. These pathways converge on the cytokine level, and thus on IL-6, the inflammatory endpoint assessed in the present study, and provide the biological rationale for examining VDR genotype as a determinant of systemic inflammation, since VDR-mediated transcriptional regulation may operate independently of circulating 25(OH)D_3_ concentrations.

In contrast to the aforementioned direct immunomodulatory 25(OH)D_3_ effects in our cohort, the inverse association of 25(OH)D_3_ and IL-6 was modest (R = −0.23, Figure 2C) and did not persist after adjustment for disease severity (Table 2), indicating that 25(OH)D_3_ behaves as a marker rather than a driver of inflammation in this context. The same applies to routine inflammatory and hematological parameters: 25(OH)D_3_ declined across disease stages while leucocyte counts and CRP rose and hemoglobin fell (Table 1), a pattern in which all four parameters track disease severity rather than reflecting a direct tested relationship with 25(OH)D_3._ Accordingly, in multivariate analysis, CRP and hemoglobin remained independent predictors of IL-6, whereas leukocytes were excluded due to collinearity (Table 2 and Table 4). This indicates that leucocyte count and CRP capture a largely shared inflammatory signal, consistent with ACLF reflecting a SIRS-like state of increased susceptibility to bacterial translocation [25,26]. The discrepancy with immunomodulatory literature may therefore reflect that, in advanced liver disease, systemic inflammation is dominated by disease-severity-related drivers that outweigh any direct immunomodulatory contribution of circulating 25(OH)D_3_, or that such effects occur at a cellular/tissue level in ways not captured by circulating IL-6. Further mechanistic studies are warranted to reconcile these observations.

Different VDR-SNPs have been described that may influence 25(OH)D_3_/VDR signaling and subsequently modulate 25(OH)D_3_ effects. Of these *TaqI*, *ApaI*, and *FokI* have been studied most in the context of various diseases [15,16,17]. Triantos et al. studied VDR-SNPs in liver cirrhosis and revealed *FokI* FF-variant to be an independent prognostic factor of survival, suggesting its potential protective role in liver cirrhosis [15]. Though, consistent data in liver cirrhosis and in particular ACLF are lacking. Our study extends this knowledge by exploring this association in a clinically highly relevant and previously underrepresented field.

We found *rs2239179* GG-variant to be associated with lower 25(OH)D_3_ levels. This points to a functional role affecting transcriptional activity in 25(OH)D_3_ metabolism that warrants further mechanistic investigation. Whether this translates into a genotype-dependent risk of acute decompensation remains open: GG genotype frequencies were comparable across compensated cirrhosis, decompensated cirrhosis and ACLF. Given the small number of ACLF patients and especially GG-genotype carriers within the ACLF group, however, this comparison was underpowered, and a genotype-dependent susceptibility to ACLF can neither be established nor excluded from our data. Adequately powered cohorts are needed to test whether GG-related 25(OH)D_3_ deficiency contributes to the inflammatory cascade precipitating ACLF.

Finally, *rs2228570 (FokI)* was nominally significantly independently associated with IL-6 levels in our cohort. A plausible mechanism is a VDR-mediated transcriptional regulation of inflammatory genes, modulated by the altered start codon and consequently shorter VDR protein of this variant [17]. Genotype-dependent differences in cytokine production have been described in cirrhosis before: Triantos et al. reported IL-1β concentrations in FokI FF carriers [15]. Direct evidence relating this variant specifically to IL-6 is nonetheless limited, and our observation—which we did not investigate functionally—therefore requires confirmation in independent cohorts.

This study has limitations. First, its single-center, observational design as well as single-timepoint sampling preclude causal interference, which is however common for genetic association studies. Associations between 25(OH)D_3,_ VDR-genotype and inflammation should not be interpreted as evidence of causality or treatment efficacy. Second, the ACLF subgroup was small and further divided by genotype, limiting statistical power. ACLF stratified comparisons should be regarded as exploratory. Third, given the number of comparisons performed across SNPs, disease stages and outcomes, individual genotype associations require independent replication and are presented as hypotheses-generating. Fourth, we did not assess VDR protein expression or downstream signaling, so that genotype-phenotype relationships remain interferential and mechanistic conclusions cannot be drawn from our findings. Lastly, information on supplementation dose, duration, adherence and timing was not systematically available, limiting interpretation of the stage-dependent supplementation findings. Overall, our findings should be regarded as hypothesis-generating and require validation in larger, prospective, multi-center cohorts with mechanistic and interventional follow-up.

## 5. Conclusions

Collectively, 25(OH)D_3_ deficiency is closely linked to cirrhosis severity and systemic inflammation. In ACLF, 25(OH)D_3_ concentrations did not differ by supplementation status. Whether this reflects reduced cellular responsiveness, which we did not assess, or differences in supplementation practice remains open and should be addressed in interventional studies. Our exploratory data further suggest that VDR genetic variation may contribute to both 25(OH)D_3_ deficiency and systemic inflammation in progressing liver cirrhosis. Future studies should aim to externally validate these findings and further elucidate mechanistic pathways.

## Figures and Tables

**Table 1:** Baseline characteristics of included patients.

	Comp. Liver Cirrhosis (N = 163)	Decomp. Liver Cirrhosis (N = 161)	ACLF (N = 31)	*p*-Value (Comp. vs. Decomp.)	*p*-Value (Comp. vs. ACLF)	*p*-Value (Decomp. vs. ACLF)
General characteristics						
Age [years], mean (SD)	52.49 (12.51)	55.88 (11.59)	57.52 (10.47)	0.1399	0.1255	>0.9999
Male gender, N (%)	83 (50.92)	93 (57.76)	22 (70.97)	0.2163	**0.0400**	0.1696
BMI [kg/m^2^], mean (SD)	27.07 (6.03)	25.02 (8.57)	26.78 (10.87)	0.2101	>0.9999	0.3813
Active alcohol consumption, N (%)	3 (1.84)	27 (16.77)	10 (32.26)	**<0.0001**	**<0.0001**	**<0.0001**
ReMELD-Na Score, mean (SD)	9.75 (3.53)	15.53 (4.55)	23.63 (6.34)	**<0.0001**	**<0.0001**	**<0.0001**
Child Pugh Score, mean (SD)	5.23 (0.7)	7.53 (1.51)	8.45 (2.05)	**<0.0001**	**<0.0001**	0.4687
25(OH)D3-supplementation, N (%)	59 (36.2)	51 (31.68)	10 (32.26)	0.3904	0.6746	0.9493
Aetiology of liver cirrhosis						
Viral, N (%)	27 (16.56)	16 (9.94)	3 (9.68)	0.0789	0.3310	0.9645
MASLD, N (%)	9 (5.52)	18 (11.18)	4 (12.9)	0.0654	0.1319	0.7827
Alcoholic, N (%)	44 (26.99)	77 (47.83)	24 (77.42)	**0.0001**	**<0.0001**	**0.0025**
Cholestatic, N (%)	28 (17.18)	18 (11.18)	0 (0)	0.1220	**0.0126**	0.0505
AIH, N (%)	49 (30.06)	31 (19.25)	0 (0)	**0.0241**	**0.0126**	**0.0004**
Complications of liver cirrhosis						
Gastrointestinal haemorrhage, N (%)	0 (0)	13 (8.07)	5 (16.13)	**0.0002**	**<0.0001**	**0.1589**
Infection, N (%)	11 (6.75)	38 (23.6)	17 (54.84)	**<0.0001**	**<0.0001**	**<0.0001**
Ascites						
Mild, N (%)	0 (0)	27 (16.77)	9 (29.03)	**<0.0001**	**<0.0001**	**0.1314**
Massive, N (%)	0 (0)	71 (44.1)	18 (58.06)	**<0.0001**	**<0.0001**	**0.0003**
Hepatic encephalopathy						
Grade I/II, N (%)	0 (0)	21 (13.04)	12 (38.71)	**<0.0001**	**<0.0001**	**0.0014**
Grade III/IV, N (%)	0 (0)	3 (1.86)	1 (3.23)	0.0800	0.0215	0.6267
Admission to ICU, N (%)	1 (0.61)	33 (20.5)	16 (51.61)	**<0.0001**	**<0.0001**	**0.0003**
Laboratory values						
Leucocytes [/nL], mean (SD)	5.18 (5.18)	6.54 (4.0)	8.93 (5.82)	0.0564	**0.0014**	0.0906
Haemoglobin [g/dL], mean (SD)	12.3 (1.91)	9.94 (2.38)	8.76 (1.7)	**<0.0001**	**<0.0001**	**0.041**
Platelets [/nL], mean (SD)	142.3 (106.1)	129.2 (99.37)	105.8 (66.77)	**0.0248**	0.1764	>0.9999
INR, mean (SD)	1.16 (0.18)	1.43 (0.36)	1.86 (0.98)	**<0.0001**	**<0.0001**	**0.0499**
CRP [mg/dL], mean (SD)	0.99 (1.44)	2.3 (2.26)	3.7 (3.77)	**<0.0001**	**<0.0001**	**0.0218**
Sodium [mmol/L], mean (SD)	138.7 (3.28)	136.0 (4.77)	134.9 (5.22)	**<0.0001**	**<0.0001**	**0.0642**
Creatinine [mg/dL], mean (SD)	1.13 (0.86)	1.06 (0.37)	2.47 (1.22)	>0.9999	**<0.0001**	**<0.0001**
Bilirubin [mg/dL], mean (SD)	1.24 (0.65)	4.2 (4.64)	10.4 (11.92)	**<0.0001**	**<0.0001**	**0.4253**
Albumin [g/dL], mean (SD)	4.43 (2.54)	3.29 (0.63)	3.19 (0.69)	**<0.0001**	**<0.0001**	>0.9999
25(OH)D_3_ [ng/mL], mean (SD)	17.83 (10.92)	13.55 (9.13)	11.27 (3.56)	**<0.0001**	**0.0068**	>0.9999
IL-6 [pg/mL], mean (SD)	2.816 (10.55)	47.46 (77.85)	75.73 (80.09)	**<0.0001**	**<0.0001**	**0.0053**

Table 1 Categorial variables are shown as N (%), continuous variables as mean ± SD. Values were calculated using the X^2^ test, Mann–Whitney Test, one-way ANOVA or Kruskal–Wallis test, as appropriate. Statistical significance was defined as *p* < 0.05. Significant *p*-values are highlighted in bold. Abbreviations: BMI, body mass index; ReMELD-Na, refitted Model of End Stage Liver Disease—Natrium; 25(OH)D_3_, 25-hydroxyvitamin D; MASLD, Metabolic Dysfunction-Associated Steatotic Liver Disease; AIH, autoimmune hepatitis; ICU, intense care unit; INR, international normalized ratio; CRP, C-reactive protein; IL-6, Interleukin-6.

**Table 2:** Univariate and multivariate linear regression analysis of predictors of IL-6 in liver cirrhosis patients.

	Univariate			Multivariate		
Parameter	β	95% CI	*p*-Value	β	95% CI	*p*-Value
**ReMELD-Na**	1.184	1.151–1.219	**<0.0001**	1.071	1.041–1.101	**<0.0001**
Age	1.031	1.014–1.049	**0.0003**	1.001	0.989–1.013	0.842
Gender	0.568	0.378–0.853	**0.007**	0.837	0.629–1.113	0.220
BMI	0.968	0.937–0.999	**0.044**	†		
Albumin	0.825	0.744–0.917	**0.0004**	‡		
CRP	1.621	1.499–1.752	**<0.0001**	1.058	1.035–1.083	**<0.0001**
Leukocytes	1.002	1.001–1.003	**0.0002**	‡		
Platelets	1.000	0.998–1.002	0.885	-		
Haemoglobin	1.013	1.002–1.025	**0.021**	0.990	0.981–0.999	**0.039**
Ascites	17.94	13.27–24.24	**<0.0001**	9.87	6.91–14.47	**<0.0001**
HE	2.394	1.293–4.437	**0.006**	‡		
25(OH)D_3_	0.965	0.946–0.984	**0.0004**	1.002	0.988–1.017	0.732
25(OH)D^3^ suppl. status	0.656	0.427–1.006	0.053	-		

Table 2 MELD, CRP, ascites and hemoglobin were independent predictors of IL-6 levels, whereas 25(OH)D_3_ did not reach significance after full adjustment. Variables with *p* > 0.05 in univariate analysis, with a high proportion of missing values (†) and collinearity (‡) were excluded from the multivariate model. Due to non-normal distribution of IL-6, it was log-transformed. Regression coefficients are presented as exponentiated values with 95% CI to facilitate interpretation on the original scale. Statistical significance was defined as *p* < 0.05 and highlighted in bold (N = 316, R^2^ = 0.589).

**Table 3:** VDR-genotype distribution did not differ significantly across disease stages.

	Comp. Liver Cirrhosis (N = 163)	Decomp. Liver Cirrhosis (N = 161)	ACLF (N = 31)	*p*-Value (Comp. vs. Decomp.)	*p*-Value (Comp. vs. ACLF)	*p*-Value (Decomp. vs. ACLF)
**Vitamin D receptor genotype**						
Haplotype (rs7968585, rs731236 (TaqI), rs7975232 (ApaI))						
wildtype (GG/TT/GG), N (%)	35 (21.4)	31 (19.25)	4 (12.9)	0.6202	0.2752	0.4016
heterozygous (GA/TC/GT), N (%)	63 (38.65)	66 (40.99)	13 (41.94)	0.6666	0.7312	0.9223
homozygous (AA/CC/TT), N (%)	18 (11.04)	19 (11.8)	3 (9.68)	0.8301	0.8225	0.7339
rs2239179						
wildtype (AA), N (%)	57 (34.97)	60 (37.27)	10 (32.26)	0.6668	0.7710	0.5957
heterozygous (AG), N (%)	84 (51.53)	81 (50.31)	19 (61.29)	0.8257	0.3184	0.2625
homozygous (GG), N (%)	22 (13.5)	20 (12.42)	2 (6.45)	0.7734	0.380	0.539
rs2228570 (FokI)						
wildtype (TT), N (%)	29 (17.79)	17 (10.56)	5 (16.13)	0.0622	0.8234	0.3726
heterozygous (TC), N (%)	69 (42.33)	79 (49.07)	13 (41.94)	0.2235	0.9674	0.4666
homozygous (CC), N (%)	65 (39.88)	65 (40.37)	13 (41.94)	0.9275	0.8304	0.8711

Table 3 Distribution of VDR-SNPs in compensated (comp.) and decompensated (decomp.) liver cirrhosis as well as in ACLF are shown as N (%). Differences were calculated using Fisher’s exact and X^2^ test, as appropriate. Corresponding *p*-values are shown in the table.

**Table 4:** Multivariate linear regression analysis of predictors of IL-6 including VDR genotype in liver cirrhosis patients.

	Univariate			Multivariate		
Parameter	β	95% CI	*p*-Value	β	95% CI	*p*-Value
**ReMELD-Na**	1.184	1.151–1.219	**<0.0001**	1.075	1.046–1.106	**<0.0001**
Age	1.031	1.014–1.049	**0.0003**	0.999	0.987–1.011	0.885
Gender	0.568	0.378–0.853	**0.007**	0.839	0.632–1.117	0.229
CRP	1.621	1.499–1.752	**<0.0001**	1.065	1.042–1.09	**<0.0001**
Haemoglobin	1.013	1.002–1.025	**0.021**	0.988	0.978–0.997	**0.012**
Ascites	17.94	13.27–24.24	**<0.0001**	9.694	6.774–13.873	**<0.0001**
25(OH)D_3_	0.965	0.946–0.984	**0.0004**	1.001	0.987–1.015	0.909
7968585/rs731236/rs7975232						
GA/TC/GT vs. GG/TT/GG	0.973	0.592–1.598	0.9125	-		
AA/CC/TT vs. GG/TT/GG	1.372	0.694–2.712	0.3609	-		
rs2239179						
AG vs. AA	1.141	0.758–1.715	0.5263	-		
GG vs. AA	0.942	0.503–1.761	0.8504	-		
rs2228570 (FokI)						
TC vs. TT	1.516	1.008–2.281	**0.0458**	1.685	1.102–2.576	**0.016**
CC vs. TT	0.878	0.579–1.331	0.5405	-		

Table 4 MELD, CRP, ascites and hemoglobin were independent predictors of IL-6 levels. Heterozygous rs2228570 carriers showed independently higher IL-6 levels compared to wildtype carriers, whereas 25(OH)D_3_ again did not reach significance after full adjustment. Variance inflation factors did not indicate relevant multicollinearity. Due to non-normal distribution of IL-6, it was log-transformed. For SNP analyses, wildtype homozygous individuals served as a reference. Regression coefficients are presented as exponentiated values with 95% CI to facilitate interpretation on the original scale. Statistical significance was defined as *p* < 0.05 and highlighted in bold (N = 316, R^2^ = 0.604).

## Data Availability

The data presented in this study are available on reasonable request from the corresponding author due to privacy reasons.

## Supplementary Materials

The following supporting information can be downloaded at https://www.mdpi.com/article/10.3390/nu18193234/s1, Table S1: Genotype frequencies, allele frequencies, Hardy–Weinberg equilibrium analysis and linkage dise-quilibrium of investigated VDR-SNPs; Table S2: Univariate and multivariate linear regression analysis of predictors of 25(OH)D_3_ in patients with liver cirrhosis.

## Abbreviations

The following abbreviations are used in this manuscript:

25(OH)D_3_	25-hydroxyvitamin D3
ACLF	Acute-on-chronic liver failure
CI	Confidence interval
CLIF-EASL	Chronic Liver Failure–European Association for the Study of Liver
Comp	Compensated
Decomp	Decompensated
DMSO	Dimethyl-sulfoxide
DNA	Deoxyribonucleic acid
ELISA	Enzyme-linked immunosorbent assay
FCS	Fetal calf serum
HC	Healthy control
HSC	Hepatic stellate cells
IL-6	Interleukin-6
LD	Linkage disequilibrium
ReMELD-Na	Refitted model of end stage liver disease—Natrium
mRNA	Messenger ribonucleic acid
PBMCs	Peripheral blood mononuclear cells
PBS	Phosphate buffered saline
SNPs	Single nucleotide polymorphisms
T_H_ cell	T helper cell
T_reg_	Regulatory T cell
VDR	Vitamin D receptor

## References

[1] Devarbhavi H., Asrani S.K., Arab J.P., Nartey Y.A., Pose E., Kamath P.S. (2023). Global burden of liver disease: 2023 update. J. Hepatol..

[2] Lee H., Kim B.K. (2022). Real-world clinical features, health-care utilization, and economic burden in decompensated cirrhosis patients: A national database. J. Gastroenterol. Hepatol..

[3] Trebicka J., Fernandez J., Papp M., Caraceni P., Laleman W., Gambino C., Giovo I., Uschner F.E., Jimenez C., Mookerjee R. (2020). The PREDICT study uncovers three clinical courses of acutely decompensated cirrhosis that have distinct pathophysiology. J. Hepatol..

[4] Arroyo V., Moreau R., Jalan R. (2020). Acute-on-Chronic Liver Failure. N. Engl. J. Med..

[5] Sarin S.K., Choudhury A., Kumar A., Mahmud N., Lee G.H., Ning Q., Tan S.-S., Thanapirom K., Arora V., Nakayama N. (2026). Acute-on-chronic liver failure: Pathophysiological mechanisms and clinical management. Nat. Rev. Gastroenterol. Hepatol..

[6] Holick M.F. (2007). Vitamin D deficiency. N. Engl. J. Med..

[7] Stokes C.S., Volmer D.A., Grünhage F., Lammert F. (2013). Vitamin D in chronic liver disease. Liver Int..

[8] Kubesch A., Quenstedt L., Saleh M., Rüschenbaum S., Schwarzkopf K., Martinez Y., Welsch C., Zeuzem S., Welzel T.M., Lange C.M. (2018). Vitamin D deficiency is associated with hepatic decompensation and inflammation in patients with liver cirrhosis: A prospective cohort study. PLoS ONE.

[9] Prietl B., Treiber G., Pieber T.R., Amrein K. (2013). Vitamin D and immune function. Nutrients.

[10] Bikle D.D., Feingold K.R., Adler R.A., Ahmed S.F., Anawalt B., Blackman M.R., Chrousos G., Corpas E., de Herder W.W., Dhatariya K., Dungan K. (2000). Vitamin D: Production, Metabolism, and Mechanism of Action. Endotext.

[11] Triantos C., Aggeletopoulou I., Thomopoulos K., Mouzaki A. (2021). Vitamin D-Liver Disease Association: Biological Basis and Mechanisms of Action. Hepatology.

[12] Beilfuss A., Sowa J.P., Sydor S., Beste M., Bechmann L.P., Schlattjan M., Syn W.-K., Wedemeyer I., Mathé Z., Jochum C. (2015). Vitamin D counteracts fibrogenic TGF-β signalling in human hepatic stellate cells both receptor-dependently and independently. Gut.

[13] Ding N., Yu R.T., Subramaniam N., Sherman M.H., Wilson C., Rao R., Leblanc M., Coulter S., He M., Scott C. (2013). A vitamin D receptor/SMAD genomic circuit gates hepatic fibrotic response. Cell.

[14] Fakhoury H.M.A., Kvietys P.R., AlKattan W., Anouti F.A., Elahi M.A., Karras S.N., Grant W.B. (2020). Vitamin D and intestinal homeostasis: Barrier, microbiota, and immune modulation. J. Steroid Biochem Mol. Biol..

[15] Triantos C., Aggeletopoulou I., Kalafateli M., Spantidea P.I., Vourli G., Diamantopoulou G., Tapratzi D., Michalaki M., Manolakopoulos S., Gogos C. (2018). Prognostic significance of vitamin D receptor (VDR) gene polymorphisms in liver cirrhosis. Sci. Rep..

[16] Levin G.P., Robinson-Cohen C., de Boer I.H., Houston D.K., Lohman K., Liu Y., Kritchevsky S.B., Cauley J.A., Tanaka T., Ferrucci L. (2012). Genetic variants and associations of 25-hydroxyvitamin D concentrations with major clinical outcomes. Jama.

[17] Cho Y.A., Lee J., Oh J.H., Chang H.J., Sohn D.K., Shin A., Kim J. (2018). Vitamin D receptor FokI polymorphism and the risks of colorectal cancer, inflammatory bowel disease, and colorectal adenoma. Sci. Rep..

[18] Martinekova P., Obeidat M., Topala M., Váncsa S., Veres D.S., Zolcsák Á., Pál M., Földvári-Nagy L., Banovcin P., Erőss B. (2025). Role of Vitamin D Supplementation in Chronic Liver Disease: A Systematic Review and Meta-Analysis of Randomized Controlled Trials. Nutr. Rev..

[19] Moreau R., Jalan R., Gines P., Pavesi M., Angeli P., Cordoba J., Durand F., Gustot T., Saliba F., Domenicali M. (2013). Acute-on-chronic liver failure is a distinct syndrome that develops in patients with acute decompensation of cirrhosis. Gastroenterology.

[20] Holick M.F., Binkley N.C., Bischoff-Ferrari H.A., Gordon C.M., Hanley D.A., Heaney R.P., Murad M.H., Weaver C.M. (2011). Evaluation, treatment, and prevention of vitamin D deficiency: An Endocrine Society clinical practice guideline. J. Clin. Endocrinol. Metab..

[21] Kitson M.T., Roberts S.K. (2012). D-livering the message: The importance of vitamin D status in chronic liver disease. J. Hepatol..

[22] Nejentsev S., Godfrey L., Snook H., Rance H., Nutland S., Walker N.M., Lam A.C., Guja C., Ionescu-Tirgoviste C., Undlien D.E. (2004). Comparative high-resolution analysis of linkage disequilibrium and tag single nucleotide polymorphisms between populations in the vitamin D receptor gene. Hum. Mol. Genet..

[23] Triantos C., Kalafateli M., Aggeletopoulou I., Diamantopoulou G., Spantidea P.I., Michalaki M., Vourli G., Konstantakis C., Assimakopoulos S.F., Manolakopoulos S. (2020). Vitamin D-related immunomodulation in patients with liver cirrhosis. Eur. J. Gastroenterol. Hepatol..

[24] Konstantakis C., Tselekouni P., Kalafateli M., Triantos C. (2016). Vitamin D deficiency in patients with liver cirrhosis. Ann. Gastroenterol..

[25] Clària J., Stauber R.E., Coenraad M.J., Moreau R., Jalan R., Pavesi M., Amorós À., Titos E., Alcaraz-Quiles J., Oettl K. (2016). Systemic inflammation in decompensated cirrhosis: Characterization and role in acute-on-chronic liver failure. Hepatology.

[26] Engelmann C., Zhang I.W., Clària J. (2025). Mechanisms of immunity in acutely decompensated cirrhosis and acute-on-chronic liver failure. Liver Int..

[27] Zhao R., Zhang W., Ma C., Zhao Y., Xiong R., Wang H., Chen W., Zheng S.G. (2021). Immunomodulatory Function of Vitamin D and Its Role in Autoimmune Thyroid Disease. Front Immunol..

[28] Mora J.R., Iwata M., von Andrian U.H. (2008). Vitamin effects on the immune system: Vitamins A and D take centre stage. Nat. Rev. Immunol..

[29] von Essen M.R., Kongsbak M., Schjerling P., Olgaard K., Odum N., Geisler C. (2010). Vitamin D controls T cell antigen receptor signaling and activation of human T cells. Nat. Immunol..

