# Supplementary material for: Vitamin D Receptor Polymorphisms Are Associated with 25(OH)D3 Concentrations and Systemic Inflammation in Decompensated Cirrhosis
Source: Nutrients. 2026 Sep 30;18(19):3234. doi: 10.3390/nu18193234 (PMC13666896; doi:10.3390/nu18193234)
Supplement: Supplementary file 1 [file nutrients-18-03234-s001.zip › nutrients-4559242-supplementary.pdf]

**Supplementary Table S1.** Genotype frequencies, allele frequencies, Hardy–Weinberg equilibrium analysis and linkage dise-quilibrium of investigated VDR-SNPs.

| | rs7968585 | rs731236 | rs7975232 | rs2239179 | rs2228570 |
|---|---|---|---|---|---|
| genotype frequency [%] | | | | | |
| AA | 22.29 | 38.22 | 20.06 | 36.62 | 15.29 |
| aA | 60.83 | 50.64 | 60.51 | 50.96 | 43.31 |
| aa | 16.88 | 11.15 | 19.43 | 12.42 | 41.4 |
| allele frequency [%] | | | | | |
| AA | 0.53 | 0.64 | 0.5 | 0.62 | 0.37 |
| aa | 0.47 | 0.36 | 0.5 | 0.38 | 0.63 |
| Hardy-Weinberg-Equilibrium | | | | | |
| X^2 | 15.21 | 2.7 | 13.87 | 2.13 | 1.55 |
| p | 0.0001 | 0.1 | 0.0002 | 0.1436 | 0.2125 |

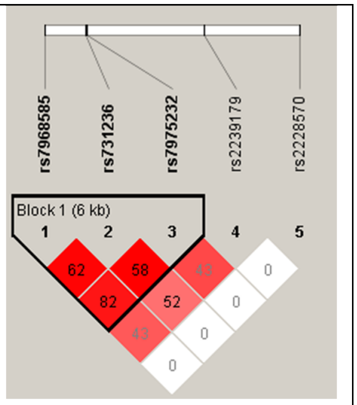


Supplementary Table S1 Genotype frequencies and allele frequencies of the investigated VDR-SNPs are shown for the study cohort. Hardy–Weinberg equilibrium was assessed using $X^2$ test. Linkage disequilibrium patterns and haplotype block structure were analysed and visualized using Haploview Software V4.2. Two variants (rs7968585 and rs7975232) of the haplotype block deviated from Hardy–Weinberg equilibrium with an excess of heterozygotes, which may reflect genotyping-related effects for these assays or the disease-selected nature of our cohort. The variants underlying the associations reported here were however in equilibrium.

**Supplementary Table S2.** Univariate and multivariate linear regression analysis of predictors of 25(OH)$D_3$ in patients with liver cirrhosis.

| | Univariate | | | Multivariate | | |
|---|---|---|---|---|---|---|
| **Parameter** | **β** | **95%CI** | ***p*-Value** | **β** | **95%CI** | ***p*-Value** |
| ReMELD-Na | 0.990 | 0.981–1.001 | 0.062 | | - | |
| 25(OH)$D^3$ suppl. status | 1.605 | 1.427–1.80 | **<0.0001** | 1.497 | 1.297–1.729 | **<0.001** |
| Age | 0.994 | 0.988–0.998 | **0.014** | 0.996 | 0.99–1.002 | 0.196 |
| Gender | 1.058 | 0.937–1.195 | 0.359 | | - | |
| BMI | 1.002 | 0.99–1.01 | 0.639 | | - | |
| Albumin | 1.024 | 0.989–1.062 | 0.182 | | - | |
| IL-6 | 0.803 | 0.713–0.904 | **0.0003** | 0.996 | 0.949–1.045 | 0.867 |
| Ascites | 0.681 | 0.603–0.769 | **<0.0001** | 0.747 | 0.609–0.917 | **0.005** |
| 7968585/rs731236/rs7975232 | | | | | | |
| GA/TC/GT vs. GG/TT/GG | 1.18 | 1.02–1.366 | **0.026** | 1.129 | 0.96–1.409 | 0.279 |
| AA/CC/TT vs. GG/TT/GG | 0.795 | 0.651–0.97 | **0.024** | 1.093 | 0.976–1.523 | 0.597 |
| Rs2239179 | | | | | | |
| AG vs. AA | 1.092 | 0.97–1.232 | 0.149 | | - | |
| GG vs. AA | 0.792 | 0.66–0.95 | **0.012** | 0.705 | 0.498–0.998 | **0.049** |
| Rs2228570 | | | | | | |
| TC vs. TT | 0.973 | 0.862–1.099 | 0.658 | | - | |
| CC vs. TT | 1.049 | 0.928–1.186 | 0.445 | | - | |

Supplementary Table S2 25(OH)$D_3$ supplementation and ascites as well as carrying GG-variant of rs2239179 were independent predictors of 25(OH)$D_3$ levels. Variables with $p < 0.05$ in univariable analysis were included in the multivariate model. Variance inflation factors of all variables did not indicate relevant multicollinearity. Due to non-normal distribution of 25(OH)$D_3$, it was log-transformed. Regression coefficients are presented as exponentiated values with 95%CI to facilitate interpretation in the original scale. Statistical significance was defined as $p < 0.05$ and highlighted in bold (N = 338), $R^2 = 0.191$).
